# Demographic, knowledge, attitudinal, and accessibility factors associated with uptake of cervical cancer screening among women in a rural district of Tanzania: Three public policy implications

**DOI:** 10.1186/1471-2458-12-22

**Published:** 2012-01-10

**Authors:** Frida S Lyimo, Tanya N Beran

**Affiliations:** 1 Weill Bugando University College of Health Sciences, P.O. Box 1464, Mwanza, Tanzania; 2 Community Health Sciences, University of Calgary, 3330 Hospital Drive NW, Calgary, AB, Canada T2N 4 N1

**Keywords:** Public health, Policy, Cervical cancer screening, Women's health

## Abstract

**Background:**

Cervical cancer is an important public health problem worldwide, which comprises approximately 12% of all cancers in women. In Tanzania, the estimated incidence rate is 30 to 40 per 100,000 women, indicating a high disease burden. Cervical cancer screening is acknowledged as currently the most effective approach for cervical cancer control, and it is associated with reduced incidence and mortality from the disease. The aim of the study was to identify the most important factors related to the uptake of cervical cancer screening among women in a rural district of Tanzania.

**Methods:**

A cross sectional study was conducted with a sample of 354 women aged 18 to 69 years residing in Moshi Rural District. A multistage sampling technique was used to randomly select eligible women. A one-hour interview was conducted with each woman in her home. The 17 questions were modified from similar questions used in previous research.

**Results:**

Less than one quarter (22.6%) of the participants had obtained cervical cancer screening. The following characteristics, when examined separately in relation to the uptake of cervical cancer screening service, were significant: husband approval of cervical cancer screening, women's level of education, women's knowledge of cervical cancer and its prevention, women's concerns about embarrassment and pain of screening, women's preference for the sex of health provider, and women's awareness of and distance to cervical cancer screening services. When examined simultaneously in a logistic regression, we found that only knowledge of cervical cancer and its prevention (OR = 8.90, 95%CI = 2.14-16.03) and distance to the facility which provides cervical cancer screening (OR = 3.98, 95%CI = 0.18-5.10) were significantly associated with screening uptake.

**Conclusions:**

Based on the study findings, three recommendations are made. First, information about cervical cancer must be presented to women. Second, public education of the disease must include specific information on how to prevent it as well as screening services available. Third, it is important to provide cervical cancer screening services within 5 km of where women reside.

## Background

Cervical cancer comprises 20 to 25% of all cancers among women in sub-Sahara Africa, which is about double that of women worldwide [1]. Its incidence in sub-Saharan countries ranges from 30 to 40 per 100,000 women [2]. In particular, cervical cancer ranks as the first most frequent form of cancer among women in Tanzania [3]. Despite considerable understanding about its etiology, currently there are no national cervical cancer service policy guidelines in Tanzania. Screening is a universally accepted early detection strategy; yet, the utilization of screening in many developing countries is still poor [4]. Although some reasons for poor uptake have been studied, the most significant ones that can directly inform public policy are not yet known. This study will simultaneously examine multiple factors to determine which are most associated with the uptake of cervical cancer screening among women sampled from a rural district in Tanzania.

### Tanzania

Tanzania has a population of 10.97 million women ages 15 years and older who are at risk of developing cervical cancer [5]. Current estimates indicate that every year 6241 of these women are diagnosed with cervical cancer and 4355 die from the disease [5]. There are many factors related to the development of cervical cancer. These include infection with high-risk HPV types 16 and 18, early sexual debut, high parity, multiple sexual partners and co-infection with HIV. Also Chlamydia trachomatis, herpes simplex virus type-2, immunosuppressant, and certain dietary deficiencies are known as risk factors for HPV infection [4]. Moreover HIV increases the incidence rates of cervical cancer, where several studies have shown a strong association between HIV-1 and invasive cancer of the cervix [6]. Although prevention in the form of HPV vaccine is shown to be effective against HPV types 16 and 18, and low risk types 6 and 11, the vaccine is expensive and generally unavailable in low income countries [6]. The World Health Organization (WHO) recommended a preventive approach of cervical cancer screening, involving a defined referral system for diagnosis, treatment and follow up [7].

Improving the screening rate is critical for early treatment and mortality reduction of cervical cancer. One means of screening is the Papanicolaou (Pap) cytology cervical scrape smear, which detects pre-cancerous lesions in the uterine cervix [8]. It has been linked to a decrease in both the incidence and mortality rates of cervical cancer in several countries [9,10]. Cytology screening, however, has been difficult to conduct in many developing countries because of the need for cytotechnologists, tracking of multiple screening visits, and specialized equipment. To overcome these difficulties, a Pap smear replaced with a simple visual screening method such as visual inspection after application of an acetic acid solution (VIA) or Lugols iodine solution (VILI) are conducted in developing countries.

### Factors related to cervical cancer screening uptake

The uptake of cervical cancer screening in many developing countries such as Tanzania is still poor due to various factors. Demographic characteristics include education, age, and marital status. In regards to education level, several studies have found that women with high screening rates have a high level of education [11,12]. However, women with high education may not necessarily seek screening [1]; thus, additional factors must be considered. Rates of screening are substantially lower in younger women aged 20-29 years and elderly women aged 60 years and above [11,13]. Also, unmarried and widowed women are less likely than married women or women living with a partner to obtain screening [11]. However, some studies have found that single women are more likely than married women to have pap screening [13,14]. Perhaps negative attitudes among the male partners, who may serve as key decision-makers, prevent women from seeking screening services [14]. The man's role may, thus, be important to take into consideration to determine whether women will access screening.

Women's knowledge is also implicated in screening uptake. Women with low levels of knowledge about cervical cancer and its prevention are unlikely to access screening services [1,11,15]. Although awareness may be a significant factor, some women, nevertheless, do not seek screening. For example, only 18% of female health workers (who were aware of the Pap smear) had actually accessed it [13]. It is likely, therefore, that awareness in combination with other factors will determine whether women utilize screening services.

In addition to knowledge, women's attitudes toward screening may be relevant. In Thailand, researchers found that female sex workers with negative attitudes about Pap smear services were less likely to have ever had a cervical smear taken than those with a positive attitude [16]. Also, in Somalia women developed a negative outlook on screening due to embarrassment associated with female genital mutilation [17]. Other cultural barriers may lead to negative opinions about screening including concern about exposure of private body parts [15]. The sex of the health worker who performs the Pap test, therefore, may be important as women may prefer one who is female [18].

Accessibility has also been identified in the research. Long distances to the cervical cancer screening services reduce the likelihood of women accessing screening [19]. A cross-sectional, community-based survey revealed that poor transportation is an additional problem [18].

In summary, cervical cancer is the leading cause of cancer mortality among women in developing countries [20]. Despite unprecedented opportunities for efficient and inexpensive prevention in the form of cervical screening, which is particularly suitable for low-resource areas, screening services are under-utilized [4]. Studies have documented the importance of the aforementioned factors separately in relation to poor service uptake, but these have yet to be examined simultaneously to determine which ones are most closely related to uptake. This information is sorely needed to inform policy development in Tanzania and identify where and how to allocate resources in an effort to significantly increase cervical cancer screening uptake. Specifically, from a randomly selected sample of women living in Moshi rural district, this study will determine (1) the rate of usage of cervical cancer screening, and (2) which of the demographic, knowledge, attitudinal and accessibility factors are most strongly related to uptake of screening services.

## Methods

### Sample

The sample was drawn from the Moshi rural Kilimanjaro region in Tanzania, where there are two centres that provide screening services targeted for women of reproductive age. Moshi rural is among the seven districts of the Kilimanjaro Region of Tanzania. It is bordered to the north by the Rombo District, to the west by the Hai District, to the east by Mwanga District and to the south by Manyara Region. According to the 2002 Tanzania national census, the population of the Moshi Rural District was 402,431 [21]. This rural area was selected for the study because it is one of the catchment areas for Mawezi Hospital and Kilimanjaro Christian Medical Center Reproductive Health Centre for cervical cancer screening services for women ages 18 to 69 years.

A multistage sampling technique was used because we wished to sample a diverse population within a large geographical area. At the district level, three wards were selected by using simple random sampling from 31 wards of Moshi rural district. From each of the three wards four villages were randomly selected for a total of 12 villages. For each village, 31 households were randomly selected, from which a woman between the ages of 18 to 69 years was randomly selected. A total of 354 of the 361 women who were invited to participate did so, resulting in a response rate of 98.1%. This number provides sufficient power to determine significance [22].

### Procedures

Research assistants, with a diploma in nursing and who participated in a day of research planning, administered a questionnaire to participants individually in their homes. Administration time was approximately one hour. The 17 questions, which are based on similar questions used in past research, were presented in four sections. The first section included four demographic questions and two questions asking if and when they had been screened for cervical cancer. The second listed five knowledge items, (e.g., "Please indicate what causes cervical cancer"). Respondents were asked to circle the correct responses. Scores were computed by taking the sum of correct responses, resulting in scores ranging from 0 to 15. Scores of 0 to 5 were considered a low level of knowledge, 6 to 10 were medium, and 11 and above were high. The third section asked four questions about attitudes towards cervical cancer screening (e.g., "Please indicate how embarrassing it is to have a cervical cancer screening.") Responses were coded on a 5-point scale from strongly disagree (1) to strongly agree (5). The fourth section listed two questions about accessibility to the nearest cancer screening center such as, "How far is the nearest facility that provides cervical cancer screening services?" The three options included, "2-5 km", "6-10 km", "more than 10 km". The questionnaire was translated from English into Kiswahili language (Tanzania's national language) so that interviews were conducted in a language which they comprehend. Then backward translation from Kiswahili to English was performed to verify the accuracy of the translation. It was then piloted in Moshi urban district, which is one of the catchment areas for cervical cancer screening services within Kilimanjaro. Problematic questions were identified and modified.

Ethics approval was obtained at Weil Bugando University College of Health Sciences, and permission was obtained from the District Executive Director of Moshi rural. Participants provided written informed consent before participating in the study.

## Results

The demographic characteristics of the participants are shown in Table 1. The age of the women was between 18 to 67 years (*M *= 38.57, *SD *= 11.9). Most were married, and almost one third had more than four children. Also, more than half had primary school level education. Less than a quarter of the participants had screened for cervical cancer, of which only about a third had done so within the last year. In addition, almost two thirds screened once, and about a third screened twice.

**Table 1:** Demographic characteristics of respondents (n = 354)

Characteristics	Frequency	Percentage
**Age (years)**		

18 - 39	191	54.0

40 - 59	149	42.1

60 +	14	4.0

**Marital status**		

Married	256	72.3

Unmarried	64	18.1

Widowed	34	9.6

**Level of education**		

None	21	5.9

Primary	196	55.4

Secondary	126	35.6

College	11	3.1

**Number of Children**		

None	53	15.0

One	33	9.3

Two	68	19.2

Three	54	15.3

Four	38	10.7

Five or more	108	30.5

**Screened**		

No	274	77.4

Yes	80	22.6

Within last year	24	30.0

Not within last year	56	70.0

Screened once	50	62.5

Screened twice	30	37.5

The relationship between screening and uptake factors are examined next. Given that 12 comparisons were conducted, the Bonferonni correction procedure was used. Thus, the probability value was lowered from 0.05 to 0.004. This criterion was used to evaluate significance. Results for this section are shown in Table 2.

**Table 2:** Relationship between Factors and Screening

	Screened			Not Screened			
	**N**	**Mean**	**SD**	**N**	**Mean**	**SD**	**Significance**

**Husband approval**	64	3.97	1.43	192	3.01	1.29	F = 24.89 p < 0.01

**Embarrassment**	80	2.54	1.34	274	3.04	1.31	F = 9.11 p < 0.01

**Painful**	80	2.40	1.25	274	3.27	1.30	F = 27.98 p < 0.001

	N	%		N	%		

**Education**							

None	3	14.3		18	85.7		Fisher's exact = 1.03
	
Primary	9	4.6		187	95.4		p < 0.001

Secondary	56	44.4		70	55.6		

College	10	90.9		1	9.1		

**Knowledge**							

High	51	75.0		17	25.0		χ2 = 1.33
	
Medium	6	8.0		69	92.0		
	
Low	23	10.4		188	89.6		p < 0.001

**Preference of provider sex**							

Male	6	7.5		37	13.5		χ2 = 10.84
	
Female	22	27.5		116	42.3		
	
Either	52	65.0		121	44.2		p < 0.01

**Awareness of center (2 missing)**							

Yes	80	34.0		155	66.0		χ2 = 52.34
	
No	0	0.0		119	100.0		p < 0.001

**Distance to facility**							

2 to 5 km	38	38.0		6	62.0		χ2 = 19.99
	
6 to 10 km	34	16.2		177	83.8		
	
> = 30 km	6	15.0		34	85.0		p < 0.001

### Demographic characteristics

An analysis of variance (ANOVA) and chi square analysis indicated that neither age nor marital status was related to screening status, *p *> 0.05. Partners' attitudes, however, were significant.

That is, women who reported higher approval for screening by their husbands were more likely to have been screened compared to those who had not screened. Also, fewer participants with secondary education or less had screened for cervical cancer compared to those with college education.

### Women's knowledge

Using the sum of the knowledge items, we determined that over half (*n *= 211, 59.6%) of the participants had a low level of knowledge of cervical cancer and its prevention, less than a quarter (*n *= 75, 21.2%) had a medium level, and less than a quarter (*n *= 68, 19.2%) had a high level of knowledge. Of the 80 women who reported having been screened, those with the highest level of knowledge about cervical cancer and its prevention were more likely than those with low and medium levels of knowledge to have been screened.

### Women's attitudes

Women who had been screened were less likely to report embarrassment than those who had not screened. Also, women who had screened were less likely to consider it painful than women who had not screened. In addition, most participants who were screened had no preference for a male or female provider compared to those not screened. Fear of receiving cervical cancer screening results, and concern about risk did not differ between those who had, or had not, screened, *p *> 0.05.

### Accessibility

Women who knew the location of the nearest cancer screening facility were more likely to have screened for cervical cancer compared to those who did not. Further, participants who resided 2 to 5 km from the cancer screening facility were more likely to have had the screening compared to those who lived further away

A logistic regression analysis was performed using the eight characteristics found to be significantly related to screening uptake (husband approval of cervical cancer screening, level of education, knowledge of cervical cancer and its prevention, concerns about embarrassment and pain of screening, preference for the sex of health provider, and awareness of and distance to cervical cancer screening services) to determine which are most closely related to screening status. As shown in Table 3 the likelihood of screening was almost nine times (OR = 8.90,95%CI = 2.139- 16.025) as likely for women who had a high level of knowledge about cervical cancer as those with less knowledge. Also, the likelihood of screening was almost four times (OR = 3.98, 95%CI = 0.180- 5.104) as high if they resided 2 to 5 km from the facility which provides cervical cancer screening services compared to those who lived further away. In sum, the most important factors related to uptake of cervical cancer screening were knowing about cervical cancer and its prevention, and residing within a five kilometers from the nearest service facility.

**Table 3:** Logistic regression results of the uptake of cervical cancer screening services among women, by selected barriers

Characteristics	*N*	Odds Ratio	(95%CI )	*P*
**Husband approval of cervical cancer screening**				
Strongly approves	69	0.57	(0.73-4.48)	0.59
Approves a bit	38	0.78	(0.08-7.60)	0.83
Neutral	79	1.98	(0.14-27.30)	0.61
Disapproves a bit	28	0.18	(0.11-2.95)	0.23
Strongly disapproves(r)	42			
				
**Level of education**				
None(r)	21			
Primary	196	0.01	(0.00-0.70)	0.35
Secondary	126	0.08	(0.00-5.47)	0.24
College	11	0.05	(0.03-0.08)	1.00
				
**Knowledge of cervical cancer and prevention**				
High level	68	**8.90**	**(2.14-16.03)**	0.00*
Medium level	75	0.04	(0.00-1.81)	0.10
Low level(r)	211			
				
**Embarrassment to have a cervical cancer screening**				
Strongly agree(r)	57			
Agree	87	0.02	(0.00- 1.03)	1.00
Undecided	29	0.05	(0.00-2.34)	0.13
Disagree	136	0.01	(0.00-1.06)	0.05
Strongly disagree	45	1.72	(0.82-2.20)	0.82
				
**Having a Pap smear taken is painful**				
Strongly agree(r)	67			
Agree	97	1.42	(0.00-1.85)	0.99
Undecided	15	3.01	(2.01-4.08)	1.00
Disagree	144	3.77	(0.28-5.87)	0.32
Strongly disagree	31	3.18	(0.09-7.15)	0.52
				
**Preference of health care provider sex to perform cervical cancer screening**				
Male health provider(r)	43			
Female health provider	138	1.00	(0.14-7.02)	1.00
Any health provider	173	1.76	(0.30-10.19)	0.53
				
**Awareness of the center which provide the services**				
Yes	235	1.42	(0.29-9.43)	1.00
No (r)	199			
				
**Distance to the nearest facility**				
2 to 5 km	100	**3.98**	**(0.18-5.10)**	0.04*
6 to 10 km	210	1.30	(1.86-3.96)	0.08
30 km and above(r)	41			

Note: r indicates the reference category for each variable, *p < 0.05

## Discussion

Several significant findings are revealed in this study. First, less than one quarter (22.6%) of women in the rural area of Tanzania had ever screened for cervical cancer. This proportion is similar to uptake rates reported in various regions within Tanzania [11] and in Nigeria [19]. Thus, it seems that women from different districts with Eastern Africa report similar rates of cancer screening use.

Second, several factors examined individually in relation to the likelihood of screening indicate that uptake is likely when women report that their husband approves of the screening; have a college education; have a high level of knowledge about cervical cancer and how to prevent it; report little embarrassment and pain associated with the cancer screening procedure; have no preference for the sex of health provider who will screen them for cervical cancer; are aware of a nearby facility; and live within a 5 km proximity. Although marital status was not related to screening status, when husbands approved of screening, women were likely to obtain the screening. Thus, their support is an important factor to consider. In societies where men have a dominant role over their wife's health seeking decisions, men's approval becomes an integral part of the women's health behaviors. It is an important intermediate step along the path to the cervical cancer screening practices. Disapproval of the service reflects a lack of personal interest or hostility to the subject. Our findings revealed that husbands' approval of cervical cancer is strongly associated with participants' cervical cancer screening status. This is confirmed by Singh et al.'s [14] study, which showed that a negative attitude of men towards screening or treatment of cervical cancer is considered a key factor contributing to poor uptake of services. Another study done in India revealed similar results [23]. In addition, women with college education had screened for cervical cancer more often compared to their counterparts. Several other studies have found the same result [11,12]. However, a study conducted at the University of Ghana indicated that few college women screened [1]. This discrepancy can be explained by the importance of knowledge of cervical cancer. Despite their level of education, the Ghana students lacked knowledge about cervical cancer and its prevention specifically, and this factor was significant in our study.

In addition to knowledge, attitudes are related to screening status. Those women who did not have a preference for the sex of health provider were more likely to have screened for cervical cancer compared to those who preferred a female health provider. Thus, it is important that women who prefer a female health worker be assured that they can indeed access one [18]. Women who thought the test was embarrassing were also less likely to obtain screening, which has been supported in other research [15]. Perhaps it is less embarrassing to show private body parts to a female rather than a male health worker. Also, the likelihood of screening is also higher for women who did not believe that it was painful, which is consistent with other studies [17,19,23]_. _Perhaps they are more tolerant of the physical discomfort of the procedure. Finally, location was related to screening status. Women who were aware of a facility which provides cervical cancer screening service were likely to obtain screening. Similar findings were obtained in a qualitative study conducted in Sudan which revealed poor awareness of services as a significant barrier [15].

The third major finding is when these factors are examined simultaneously in relation to screening, only women's knowledge about cervical cancer and its prevention, and accessibility to a screening facility are significantly associated with screening status. In other words, when women are knowledgeable and live close to a facility, they are likely to seek screening, regardless of demographic and attitudinal factors. Understanding the importance of screening provides women with a valid reason for seeking this service. Having full information about the cervical cancer and its prevention gives women the ability to make an informed decision. It is also likely that women become knowledgeable about cervical cancer screening as a result of seeking the service. Also, the distance women have to travel to obtain this service is critical due to cost and limited access to transportation. The results are consistent with the Health Belief model, whereby those with perceived benefits are more likely to take preventive actions, than those with no perceived benefits or low perceived benefits [24].

### Limitations

This study has some limitations. First, it was conducted in the community of Moshi rural district, which may not be generalizable to other areas. Second, the method of interviewing may have influenced the results. That is, women may have responded in a positive manner to the questions to present themselves in a socially desirable way. Also, some questions may have seemed leading and perhaps influenced how women responded. Third, their perceptions of their husbands' attitudes may not be accurate. Similarly, responses are all self-report and may not reflect true events. Finally, reliability and validity of the responses were not verified; however, the instrument was pilot tested.

### Recommendations

These results have three specific implications. First, women must be informed about cervical cancer and how to prevent it. Awareness campaigns must provide accurate information so that women can make informed choices. Second, these campaigns must emphasize the importance and effectiveness of prevention in the form of regular cervical cancer checks. Thus, information is important, but must be combined with prescriptive information about how to take preventive action. Third, accessibility to screening must be improved. Findings from this study reveal that distance of the facility is a crucial determinant of whether women will access cervical cancer screening services.

## Conclusions

In conclusion, cervical cancer screening is currently recognized as the most effective approach for cervical cancer control, and it is associated with reduced incidence and mortality from the disease. This study has shown that several demographic, knowledge, attitudinal, and accessibility factors are associated with a decreased likelihood of women utilizing this screening. Although these are important to consider and policies can address all of them, it is recommended that resources specifically target those most closely associated with uptake: women's lack of knowledge about cervical cancer and its prevention, and limited access to a facility providing cancer screening. Eliminating these barriers is paramount if we want to achieve the goal of reducing the incidence and mortality rates of cervical cancer.

## Competing interests

The authors declare that they have no competing interests.

## Authors' contributions

Both authors contributed equally to this work.

FF conceived of the study and collected data. TNB advised on the design and helped write the manuscript. Both authors read and approved the final manuscript.

## Pre-publication history

The pre-publication history for this paper can be accessed here:

http://www.biomedcentral.com/1471-2458/12/22/prepub
